# Supplementary material for: A Global Screen for Assembly State Changes of the Mitotic Proteome by SEC-SWATH-MS
Source: Cell Syst. 2020 Feb 26;10(2):133–155.e6. doi: 10.1016/j.cels.2020.01.001 (PMC7042714; doi:10.1016/j.cels.2020.01.001)

Q12962 | TAF10_HUMAN | TAF10 TAF2A TAF2H TAFII30
Monomer MW [kDa]: 21.711 Monomer expected elution fraction: 54

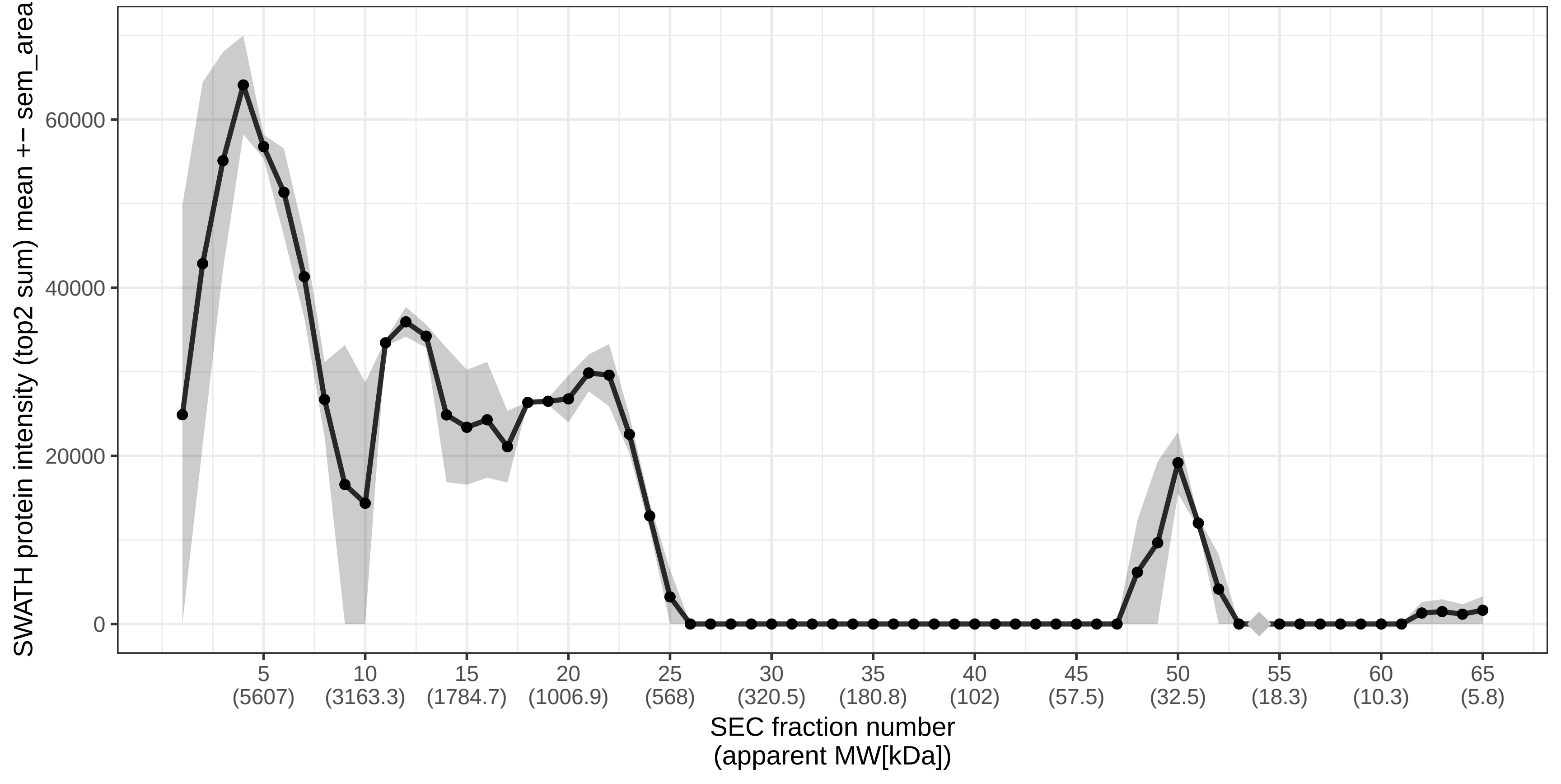

Supplement: Data S1. SEC-SWATH-MS Protein Chromatograms, Related to Figure 1 [file mmc6.zip › SECchrom_Q12962_TAF10_HUMAN_TAF10_TAF2A_TAF2H_TAFII30.pdf]
